# Prolonged Antimicrobial Effects of *Eucalyptus* Oil via C_8_‐Functionalized Silica Monolith

**DOI:** 10.1155/ijm/6874990

**Published:** 2026-06-09

**Authors:** Wimonrut Insuan, Orapin Insuan, Sirapan Sukontasing, Patamaporn Umnahanant, Thippayarat Chahomchuen

**Affiliations:** ^1^ Department of Veterinary Technology, Faculty of Veterinary Technology, Kasetsart University, Bangkok, Thailand, ku.ac.th; ^2^ Department of Medical Technology, School of Allied Health Sciences, University of Phayao, Phayao, Thailand, up.ac.th

**Keywords:** C_8_-silica monolith, delivery system, *Eucalyptus* oil release, evaporation kinetic, monolithic adsorbent, sustained antimicrobial activity

## Abstract

This study presents the synthesis and characterization of a mesoporous silica‐based monolith functionalized with octyl (C_8_) groups, developed as an absorbent for the controlled release of *Eucalyptus* oil (Eu‐oil). The C_8_‐silica monolith was prepared via a two‐step acid/base‐catalyzed sol‐gel process and characterized using various analytical techniques. Chemical profiling of the Eu‐oil and Eu‐oil‐loaded monolith was conducted using gas chromatography‐mass spectrometry (GC‐MS). Evaporation tests revealed that Eu‐oil exhibited rapid volatilization within 24 h, whereas Eu‐oil‐loaded on the C_8_‐monolith had a markedly slower release. The GC‐MS analysis confirmed that the monolith effectively retained volatile constituents, particularly eucalyptol, *β*‐myrcene, and *γ*‐terpinene, thereby extending the release of bioactive compounds. Antimicrobial assays showed that Eu‐oil delivered via the monolith maintained bacteriostatic activity against *Escherichia coli* and *Staphylococcus aureus* for up to 7 days, outperforming the rapid activity loss observed with Eu‐oil applied on filter discs. Similarly, antifungal tests against *Aspergillus* sp. isolated from garlic demonstrated prolonged suppression of fungal growth. Kinetic modeling further confirmed that the Eu‐oil release followed a concentration‐dependent, diffusion‐assisted mechanism, which was best described by the first‐order and Weibull models. These findings underscored the potential of mesoporous C_8_‐silica monoliths as efficient carriers for volatile essential oils, offering a promising strategy for sustained antimicrobial delivery in healthcare and food preservation applications.

## 1. Introduction

In recent years, there has been increased worldwide interest in the use of natural bioactive compounds, particularly essential oils (EOs), as alternatives for controlling bacterial or fungal contamination in food, agriculture, and pharmaceutical systems [1–3]. EOs are natural volatile compounds extracted from plants and usually complex mixtures of natural compounds, both polar and nonpolar, which contain around 20–60 components at quite different concentrations [4]. EOs contain broad‐spectrum antimicrobial plant‐derived metabolites that inhibit both the growth of fungi and their production of toxic metabolites, as well as having antifungal properties against pathogenic fungi [5, 6]. Among them, *Eucalyptus* oil (Eu‐oil) has attracted particular interest due to its broad‐spectrum antibacterial and antifungal activities [7, 8], making it a promising candidate for applications in food preservation [9], pharmaceutical formulations [10, 11], and veterinary medicine [12–14]. Such bioactive properties render EO valuable tools for mitigating microbial contamination and reducing foodborne and postharvest losses.

Despite their bioactivity, the practical use of EOs is limited by poor water solubility, high volatility, sensitivity to light and heat, and potential toxicity at elevated concentrations [15–17]. Therefore, it is important to develop delivery systems capable of protecting and controlling the release of EOs.

Synthetic materials have been used extensively as carriers to improve the stability and controlled release of natural extracts and EOs. Various classes of materials have been investigated, including biopolymers (chitosan, alginate, and gelatin) [18, 19], lipid‐based carriers (liposomes and solid lipid nanoparticles) [11], synthetic polymers (poly (lactic‐co‐glycolic acid) and polycaprolactone) [20], and inorganic porous carrier (silica and zeolite) [21, 22]. Among these, silica‐based materials have gained increasing attention due to their high surface area, tunable mesoporosity, chemical inertness, and ease of functionalization. Their interconnected porous networks facilitate efficient loading and sustained release of volatile compounds while protecting them from environmental degradation [23].

Silica monoliths allow precise control over pore structure and material morphology. Monolith or monolithic material can be synthesized via the sol‐gel process, involving a single piece of porous material with macropores and mesopores, which make them ideal candidates for controlled‐release applications [24–27]. Functionalization with hydrophobic groups such as octyl (C_8_) chains can further enhance oil‐matrix interactions, potentially improving retention and the release behavior of nonpolar EOs.

In this work, we report the synthesis and characterization of a mesoporous C_8_‐silica‐based monolith material designed as an absorbent for the controlled release of Eu‐oil. The release behavior was investigated for its correlation with the structural characteristics of the synthesized monolith, while the antimicrobial activity was evaluated against representative foodborne bacteria (*Escherichia coli* and *Staphylococcus aureus*) and fungal pathogens (*Aspergillus* sp.). This approach is aimed at elucidating how the silica structure influences the release function and at demonstrating the potential of monolith‐based carriers for sustained antimicrobial applications in foodborne, agricultural, and biomedical applications, with the study design focusing on evaluating the potential of volatile compounds as alternative antimicrobial agents for vapor‐phase applications.

## 2. Materials and Methods

### 2.1. Materials

In the synthesis of the C_8_‐silica monolith in 96‐well plate plastic molds, tetraethylorthosilicate (TEOS, > 99%) and triethoxyoctylsilane (C_8_‐TEOS, > 96.5%) were used as precursors, and dodecylamine (> 98%), bought from Merck (Darmstadt, Germany), and methanol (analytical reagent grade) Carlo Erba (Rodano, Italy), acted as a porogenic reagent. Hydrochloric acid (37%) from QReC (New Zealand) functioned as an acid catalyst for hydrolysis and condensation. Dodecylamine (>98%) bought from Merck (Darmstadt, Germany) was added into the solution for gel formation. The Eu‐oil used in this study was obtained from *Eucalyptus globulus* (*Eucalyptus camaldulensis)* extracted using the hydrodistillation method in our earlier study [8]. The EO had been stored in sealed amber glass vials at −20°C to prevent degradation.

### 2.2. Preparation of C_8_‐Silica Monolith

The C_8_‐silica monolithic was synthesized via a two‐step acid/base catalyzed sol‐gel process, adapted from Yan et al. [28]. The formulation mixture to produce the sol solution contained 180 *μ*L of methanol as a solvent, 90 *μ*L of TEOS, 50 *μ*L of C_8_‐TEOS, 10 *μ*L of water, and 10 *μ*L of 0.5 M HCl. The liquid mixture was kept at a constant 60°C for 5 h before adding 10 *μ*L of dodecylamine into the solution for gel formation. The solution was filled manually into 96‐well plate plastic molds to make a cylindrical form, kept at a constant 40°C for 12 h, and then washed with ethanol. Subsequently, the C_8_‐silica monolithic was incubated at 60°C for 6 h and kept in a desiccator before use.

### 2.3. Morphology Characterization and Chemical Structure

The morphology of the monolithic material was characterized using scanning electron microscopy (SEM). For the SEM observation, the dried monolithic samples were sputtered with an Au‐conductive coating for 2 min and then observed using SEM (Quanta 450; FEI; Hillsboro, Oregon, United States). The chemical structure of the monolith was analyzed using a Fourier‐transform infrared spectrometer (PerkinElmer; United States), and the FTIR spectra were recorded using transmittance mode over the wavenumber range 650–4000 cm^−1^ at a resolution of 4 cm^−1^. The Brunauer–Emmett–Teller (BET) surface area was determined using nitrogen adsorption–desorption analysis (Micromeritics Instruments; United States). Thermogravimetric analysis (TGA) was performed (TGA 8000; PerkinElmer; United States) in range 30°C–600°C at a heating rate of 5°C/min in flowing nitrogen.

### 2.4. Evaporation Test of Eu‐Oil With and Without C_8_‐Monolith

Evaporation rates of Eu‐oil were evaluated under two conditions at room temperature. In the first, 50 *μ*L of Eu‐oil was placed directly into an amber glass vial. In the second, the same volume was loaded onto a C_8_‐monolith and transferred into a vial. All samples were weighed at 24‐h intervals over 5 days. Weight loss percentages were calculated to compare evaporation between the Eu‐oil and the monolith‐adsorbed oil. Chemical compositions of Eu‐oil with and without the C_8_‐monolith were further analyzed using gas chromatography‐mass spectrometry (GC‐MS).

The evaporation behavior of the Eu‐oil from the C_8_‐monolith was evaluated using five kinetic models: zero‐order, first‐order, second‐order, Higuchi, and Weibull. These models were chosen for their relevance in describing controlled release and diffusion in porous matrices [29, 30]. Model parameters were analyzed based on regression analysis using the Excel software (Microsoft Corp.; Redmond, Washington, United States). Model performance was assessed using the coefficient of determination (*R* ^2^) and root mean square error (RMSE), with the optimal model identified based on the highest *R* ^2^ and the lowest RMSE values [29].

### 2.5. Qualitative Evaluation of Eu‐Oil Using GC‐MS

The chemical components of the two Eu‐oil samples (Eu‐oil and Eu‐oil‐loaded C_8_‐monolith) were analyzed using GC‐MS. Each Eu‐oil sample was dissolved in n‐hexane. Subsequently, 200 *μ*L of the internal standard (1000 *μ*g/mL of n‐decane) was added to the solution, mixed thoroughly, and filtered prior to the GC‐MS analysis. The gas chromatographic system was interfaced with a mass spectrometer (GC‐MS‐QP2010 Systems; Shimadzu; Japan) at 70 eV ionization energy and the EI ionization mode and equipped with a ZB‐Wax capillary column (30 m × 0.25 mm, 0.25 mm film thickness). Mass scanning was carried out in the range 40–500 amu. Helium was used as a carrier gas with a flow rate of 1.2 mL/min. The injector, GC‐MS interface, and the ion source temperatures were 250°C. The oven temperature was programmed from 60°C to 230°C at 5°C/min [8]. The injected volume was 1 *μ*L (split ratio 10: 1). Identification of compounds was based on comparisons of the mass spectra with those of the NIST mass spectral library data standards of the GC‐MS system.

### 2.6. Bacterial Strains and Culture Conditions

The standard bacterial reference strains used were *E. coli* (ATCC 25922) and *S. aureus* (ATCC 25923). All strains were maintained on Mueller–Hinton agar (MHA; Difco; United States) and subcultured in Mueller–Hinton broth (Difco; United States) prior to testing.

### 2.7. Disc Diffusion Assay and Sustained‐Release Evaluation

The antibacterial activity of the Eu‐oil was assessed using a modified Kirby–Bauer disc diffusion method to evaluate both immediate and sustained effects [8]. Bacterial suspensions (0.5 McFarland, ~1.5 × 10^8^ CFU/mL) were evenly spread on MHA plates using sterile cotton swabs. Two delivery formats were tested: (i) sterile filter paper discs (6 mm) and (ii) C_8_‐silica monoliths (5 mm), each loaded with 10 *μ*L of Eu‐oil under aseptic conditions. Each Eu‐oil‐loaded carrier was placed centrally on an inoculated agar plate, sealed with parafilm to prevent evaporation, and incubated at 37°C for 24 h. The diameter of the inhibition zone (DIZ) was measured in millimeters, excluding the carrier (disc or monolith) diameter.

For sustained‐release evaluation, the same Eu‐oil‐loaded carriers were aseptically transferred daily onto freshly inoculated MHA plates with the same bacterial strain for seven consecutive days. Dimethyl sulfoxide (DMSO)–loaded discs and monoliths served as negative controls. All experiments were performed in triplicate, and the results were expressed as mean DIZ ± standard deviation. Release profiles were compared by plotting DIZ values over time.

### 2.8. Isolation and Identification of Fungi From Garlic Samples

Fungal strains were isolated from garlic bulbs exhibiting visible fungal growth. Samples were surface‐sterilized with 70% ethanol for 1 min, and small tissue fragments (2–3 mm) from infected areas were aseptically placed on a potato dextrose agar (PDA; Himedia, India) plate. Plates were incubated at 30°C in the dark for 3–5 days. Emerging colonies were subcultured by transferring hyphal tips onto fresh PDA until pure isolates were obtained. Colony morphology (color, texture, and pigmentation) and microscopic features (hyphae and spore shape) were examined under a light microscope with lactophenol cotton blue (LPCB) staining (Sigma‐Aldrich, United States). Pure cultures were maintained on PDA slants and stored at 4°C for further experiments.

### 2.9. Antifungal Activity Assay of Eu‐Oil and Eu‐Oil Loaded on C_8_‐Monolith

The fungal isolate was subcultured prior to use. Agar plugs were excised from the actively growing margins of 3–5‐day‐old colonies using a sterile cork borer (No. 3) and aseptically transferred to the center of fresh PDA plates. For the vapor assay, C8‐monolith samples were loaded with 20 *μ*L of Eu‐oil and positioned on the inner surface of the plate lid. Plates were then inverted, sealed with parafilm to minimize evaporation, and incubated at 30°C. The vapor released from Eu‐oil inhibited fungal growth on the agar surface. Radial growth of fungal colonies was observed every 24 h. Control plates received an equal volume of DMSO on the filter disc instead of the EO. No standard antifungal agent was included, as conventional antifungal compounds are nonvolatile and not suitable for vapor‐phase assays. All experiments were conducted in triplicate [31].

## 3. Results and Discussion

### 3.1. Preparation of C_8_‐Silica Monolith

The C_8_‐monolith was successfully synthesized via a two‐step acid/base catalyzed sol‐gel process. A sol‐gel process using the C_8_‐TEOS and TEOS precursor under acidic conditions was followed by aging and drying. The resulting monoliths were white, rigid, and crack‐free, indicating successful gelation and drying without shrinkage. Photographs of samples of the C_8_‐silica monolith are shown in Figure S1. The monoliths had a cylindrical shape with uniform surface texture, suggesting good control over the synthesis parameters.

### 3.2. Morphology Characterization and Chemical Structure

**Figure 1:**
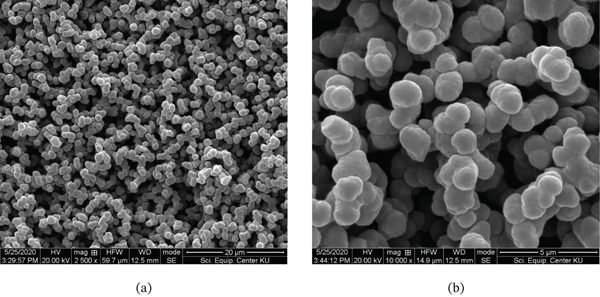
SEM image of C_8_‐silica monolith with spherical shape at different magnifications: (a) 2500× and (b) 10,000× (scanning conditions: accelerating voltage = 20 kV, working distance (WD) = 12.5 mm, detector = SE).

The surface morphology of the synthesized C_8_‐monolith was examined using SEM, as presented in Figure 1. The SEM imagery revealed an interconnected network structure, which is characteristic of typical monolithic materials synthesized via the sol‐gel method [32]. As shown in Figure 1a (low magnification), the C_8_‐monolith had a highly porous and uniform structure, composed of interconnected spherical particles, suggesting a consistent and successful sol‐gel synthesis process. The individual spherical particles and their interconnections were more clearly visible at a higher magnification (Figure 1b). These particles appeared to be agglomerates of smaller primary particles, forming a continuous network. The visible junctions between these particles confirmed the monolithic nature of the material. The interconnected pores observed in the micrograph are ideal for applications requiring efficient mass transfer, such as slow‐release systems for EOs, as the open channels allow the EO to load easily into the pores and be released slowly over time [23].

The FTIR spectrum of the synthesized C_8_‐functionalized silica monolith which displayed the characteristic absorption bands confirming surface modification of the C_8_ organic functional groups [33] is shown in Figure S2. The strong peak at ~1060 cm^−1^ corresponded to asymmetric Si–O–Si stretching [34], indicating successful silica network formation. Additional bands at ~805 cm^−1^ and a shoulder near 950 cm^−1^ represented symmetrical Si–O–Si and Si–OH stretching, respectively, suggesting the presence of residual silanol groups remaining on the surface [35, 36]. The peaks observed around 2900 cm^−1^ indicated the presence of the C–H stretching vibrations of the methyl and methylene groups, confirming successful surface modification with octyl groups [36]. The broad band around 3373 cm^−1^ indicated –OH stretching, possibly from surface silanol groups (Si–OH) or adsorbed moisture. These results confirmed the successful formation of a C_8_ modification to the mesoporous silica network.

**Figure 2:**
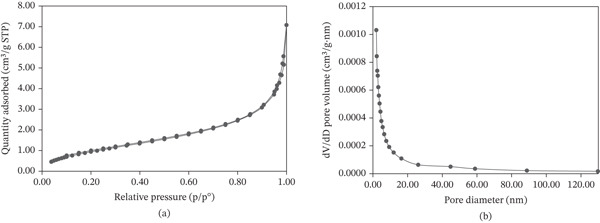
BET analysis of the C8–silica monolith: (a) nitrogen adsorption–desorption isotherm and (b) BJH desorption pore size distribution.

The surface and porosity characteristics of the synthesized C8‐functionalized silica monolith were evaluated using nitrogen adsorption–desorption analysis, with the results summarized in Table S1. The BET surface area was 3.90 m^2^g^−1^, and the total pore volume was 0.008 cm^3^g^−1^ (typical mesoporous silica powders have a range of 500–1000 m^2^g^−1^) [37–39]. The average pore diameter was in the range 7.9–8.5 nm, clearly indicating the mesoporous nature of the material. The nitrogen adsorption isotherm (Figure 2a) contained a hysteresis loop, indicative of mesopore formation, while the BJH desorption pore size distribution (Figure 2b) revealed a narrow pore distribution centered at around 8 nm. This surface modification improves compatibility with nonpolar EO molecules. Overall, these features suggested that the C_8_‐functionalized silica monolith had an appropriate balance of hydrophobicity, pore volume, and mesoporous structure for efficient adsorption and sustained release of nonpolar EOs.

**Figure 3:**
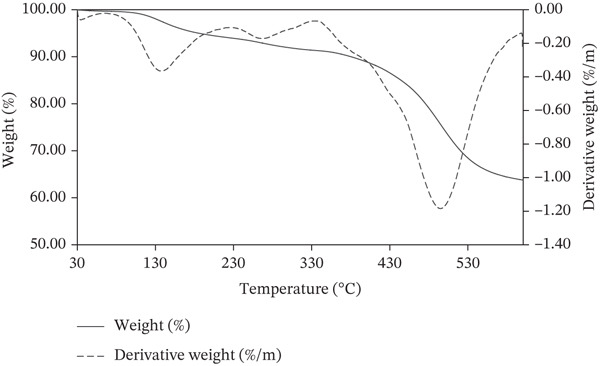
TGA curve (solid line) of C_8_‐monolith silica and its first derivative (dashed line) studied in range 30°C–600°C.

The thermal stability of the synthesized C_8_‐functionalized silica monolith was evaluated using TGA under a nitrogen atmosphere. The TGA profile (Figure 3) displayed a multistep weight loss, while the corresponding derivative thermogravimetry (DTG) curve highlighted the temperatures of maximum decomposition rates. An initial weight loss of approximately 5% in the range 30°C–150°C was attributed to the evaporation of H_2_O and residual solvent molecules from the porous silica structure, whereas the loss of organic compound usually occurs in the temperature interval 150°C–600°C [40, 41]. The most substantial weight loss occurred in the range 400°C–600°C, with a DTG peak at approximately 500°C, corresponding to the thermal degradation of covalently bonded C8 organic chains. The total weight loss up to 600°C was approximately 65%, reflecting the extent of organic functionalization.

### 3.3. Evaporation Test of Eu‐Oil With and Without C_8_‐Monolith

**Figure 4:**
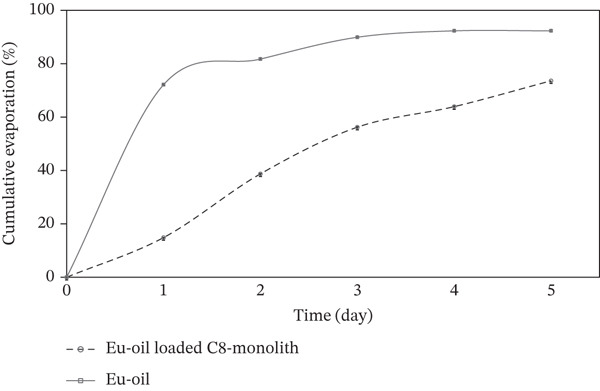
Cumulative evaporation profile over time of Eu‐oil with and without C_8_‐monolith. Data are presented as mean ± SD (*n* = 3).

Evaporation was compared of the Eu‐oil and Eu‐oil loaded onto the C_8_‐silica monolith through an in vitro assay over 5 days at room temperature, with the evaporation profiles shown in Figure 4. The Eu‐oil had a rapid evaporation rate, with approximately 70% of its weight lost within the first 24 h and reaching almost complete evaporation (> 90%) by Day 3. In contrast, the Eu‐oil loaded on the C_8_‐monolith had a much slower release pattern, with only about 15% weight loss after 24 h, and cumulative evaporation reaching 40% and 55% at Days 2 and 3, respectively. After 5 days, the total evaporation of the Eu‐oil loaded on the monolith was around 75%, still markedly lower than for the pure Eu‐oil which had already plateaued at nearly 90%. These findings demonstrated that the C_8_‐functionalized monolith effectively retarded the volatilization of Eu‐oil, confirming its ability to act as a material for the controlled release of volatile bioactive compounds.

The evaporation profile of the Eu‐oil from the C_8_‐functionalized silica monolith was analyzed using five kinetic models: zero‐order, first‐order, second‐order, Higuchi, and Weibull. Linearized equations were applied, and the corresponding slopes, intercepts, rate constants (*K*), and *R* ^2^ and RMSE values were calculated and are summarized in Table S2. Among the tested models, the first‐order model had the best fit (*R* ^2^ = 0.9922; RMSE = 0.0186), indicating that the evaporation process was concentration‐dependent, consistent with the classical kinetic behavior reported in controlled‐release systems [42]. The Weibull model also showed a strong correlation (*R* ^2^ = 0.9844; RMSE = 0.0419), with a shape parameter (*b* = 1.3) greater than 1, suggesting a non‐Fickian release mechanism [43]. The Higuchi model had a similarly high *R* ^2^ (0.9848), supporting a diffusion‐controlled process through the porous silica matrix.

**Figure 5:**
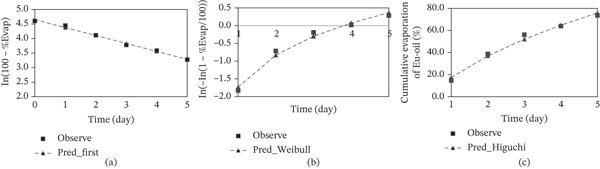
Comparison of observed and predicted Eu‐oil release profiles for (a) first‐order, (b) Weibull, and (c) Higuchi models.

Figure 5 compares the observed and predicted release profiles for the first‐order, Weibull, and Higuchi models. The close overlap between the experimental and predicted values in the first‐order and Weibull models (Figure 5a,b) further supported their suitability for describing the release behavior. Overall, these findings suggested that Eu‐oil evaporation from the C_8_‐silica monolith followed a concentration‐driven, diffusion‐assisted mechanism, providing sustained release suitable for controlled delivery applications. [29]

### 3.4. Chemical Composition of Eu‐Oil and Eu‐Oil‐Loaded Monolith Based on GC‐MS

The chemical composition of the Eu‐oil before and after adsorption onto the C_8_‐functionalized silica monolith was analyzed using GC‐MS, as summarized in Table 1. The GC‐MS chromatograms and peak identification details are provided in the Supporting Information (Figures S3 and S4). The major constituents of the original Eu‐oil included eucalyptol (24.93%), *γ*‐terpinene (18.00%), and *β*‐myrcene (20.23%). After 5 days of evaporation testing, the Eu‐oil extracted from the monolith still contained these compounds, but at reduced relative abundance eucalyptol (18.55%), *γ*‐terpinene (13.99%), and *β*‐myrcene (15.80%), indicating partial retention of the volatiles during the loading and release process. Notably, after evaporation, the highly volatile monoterpenes, such as *α*‐pinene, *β*‐pinene, and (+)‐4‐carene, were considerably reduced or undetected, while the more stable oxygenated compounds, such as eucalyptol and terpinen‐4‐ol, were preserved to a greater extent. This trend, confirmed by the GC‐MS analysis, suggested that the C_8_‐monolith selectively slowed the evaporation of volatile constituents, thereby enhancing the sustained release of bioactive components.

**Table 1:** Chemical constituent analysis of Eu‐oil with and without C_8_‐monolith by GC‐MS.

No.	RT (min)	Chemical constituent	Chemical structure	Area ratio to IS	
				Eu‐oil	Eu‐oil‐loaded C_8_‐monolith
1	3.119	*α*‐Pinene	C_10_H_16_	2.22	1.72
2	3.165	*α*‐Phellandrene	C_10_H_16_	0.74	0.61
3	4.458	*β*‐Pinene	C_10_H_16_	0.11	0.09
4	5.54	*β*‐Myrcene	C_10_H_16_	20.23	15.8
5	5.795	(+)‐4‐Carene	C_10_H_16_	1.02	0.72
6	6.147	D‐Limonene	C_10_H_16_	2.65	2.14
7	6.304	Eucalyptol	C_10_H_18_O	24.93	18.55
8	7.033	*γ*‐Terpinene	C_10_H_16_	18.00	13.99
9	7.481	o‐Cymene	C_10_H_14_	5.53	6.01
10	7.672	Isoterpinolene	C_10_H_16_	1.30	0.99
11	10.77	Citronellal	C_10_H_18_O	0.29	—
12	11.673	Linalool	C_10_H_18_O	0.2	0.10
13	11.891	p‐Menth‐2‐en‐1‐ol	C_10_H_18_O	0.18	0.11
14	12.416	Terpinen‐4‐ol	C_10_H_18_O	3.23	2.18
15	12.723	p‐Menth‐2‐en‐1‐ol	C_10_H_18_O	0.13	—
16	13.567	*γ*‐Terpineol	C_10_H_18_O	0.93	0.49
17	13.964	Isopiperitone	C_10_H_16_O	0.44	0.26
18	17.622	(‐)‐Globulol	C_15_H_26_O	0.41	0.25

*Note:* (‐) = not detected.

Abbreviations: IS = internal standard, RT = retention time.

### 3.5. Antimicrobial Activity and Sustained‐Release Properties of Eu‐Oil‐Loaded C_8_‐Monolith

**Figure 6:**
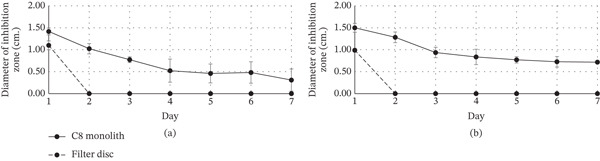
Diameter of inhibition zone measured over 7 days comparing two delivery systems of *Eucalyptus* essential oil (C8‐monolith and filter disc) for (a) *E. coli* and (b) *S. aureus*. Values represent inhibition zones excluding the diameter of the disc and monolith.

The antibacterial activity of the Eu‐oil was evaluated using two delivery systems (C_8_‐monolith and filter discs) against *E. coli* and *S. aureus*. The DIZ served as a quantitative indicator of antimicrobial activity. As shown in Figure S5, the DIZ values from the C_8_‐monolith were consistently higher than those from the filter discs throughout the 7‐day observation period. For the filter disc system, a sharp decline in inhibition was observed within the first 24 h, indicating a burst release of Eu‐oil followed by rapid volatilization and diminished antimicrobial efficacy. In contrast, the C_8_‐monolith maintained measurable inhibition zones against both *E. coli* (Figure 6a) and *S. aureus* (Figure 6b) over the entire experimental period, thereby demonstrating a more sustained antibacterial effect.

**Figure 7:**
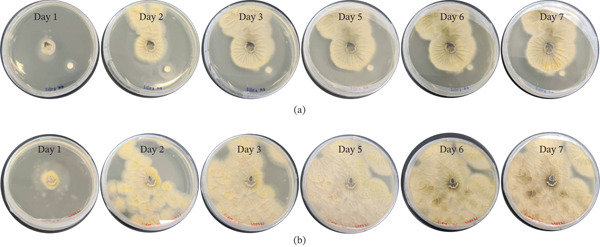
Visual observation of *Aspergillus* sp. growth inhibition on PDA over 7 days. (a) Colony morphology of the fungus treated with Eu‐oil‐loaded C_8_‐monolith. (b) Colony morphology of the fungus treated with DMSO‐loaded C_8_‐monolith (control). Each panel represents the colony morphology on the indicated day. Experiments were performed in triplicate.

The antifungal efficacy of the Eu‐oil‐loaded C_8_‐monolith was also evaluated against *Aspergillus* spp. isolated from garlic samples. The morphological and microscopic examinations confirmed the isolates as *Aspergillus*, characterized by dark gray conidia with a white border, cream coloration on the reverse colony surface, and biseriate phialides radiating in all directions (Figure S6). Visual observation of colony development on PDA over 7 days (Figure 7) revealed that the Eu‐oil‐loaded C_8_‐monolith markedly suppressed mycelial expansion, resulting in progressive inhibition of fungal growth (Figure 7a). In contrast, the DMSO‐loaded C_8_‐monolith (control) allowed unrestricted fungal proliferation and typical colony morphology (Figure 7b), confirming that the antifungal effect was attributable to the Eu‐oil release from the monolith carrier.

The superior performance of the C_8_‐monolith system compared to the filter discs can be explained by its physicochemical properties. Eu‐oil adsorption is enhanced by its hydrophobic and porous matrix that facilitates gradual desorption, which minimizes evaporation losses and enables controlled release. This mechanism would account for the extended antimicrobial activity observed. Such controlled‐release behavior was consistent with other reports showing that porous or hydrophobic carriers could stabilize EOs and prolong their bioactivity [44–46]. In addition, the prolonged antifungal activity against *Aspergillus* spp. under vapor‐phase conditions demonstrates the C_8_‐monolith system’s effectiveness in mediating noncontact inhibition through sustained release of volatile Eu‐oil components, where antimicrobial efficacy depends on compound volatility and diffusion to the microbial interface. Vapor phase often exhibits enhanced activity compared to liquid phase due to enrichment of volatile monoterpene fractions that improve diffusion and antimicrobial interaction [47]. EOs exert antifungal effects by disrupting membrane integrity, inhibiting ergosterol biosynthesis, and interfering with cell wall synthesis; accordingly, fungi generally show greater sensitivity than bacteria, particularly Gram‐negative species, due to the presence of lipopolysaccharide barriers [44, 48]. The extended growth suppression observed in the current study indicated that the C_8_‐monolith could provide a favorable release profile to maintain Eu‐oil concentrations at bioactive levels for several days. These findings highlighted the potential of mesoporous C_8_‐silica monoliths as effective carriers for volatile EOs, offering a promising platform for long‐term antimicrobial applications in healthcare and food preservation.

## 4. Conclusion

The C_8_‐functionalized silica monolithic materials were successfully synthesized via a sol‐gel process using TEOS and C_8_ organosilanes. The resulting monoliths were rigid and crack‐free and exhibited a uniform mesoporous structure composed of interconnected spherical particles, confirming successful surface functionalization. The C_8_‐silica monolith demonstrated effective adsorption and sustained release of the Eu‐oil, with release kinetics consistent with a concentration‐driven, diffusion‐assisted mechanism. Its hydrophobic and porous structure facilitated strong interactions with volatile and nonpolar components, thereby retarding evaporation and prolonging bioactive release.

These findings highlighted the potential of C8‐functionalized silica monoliths as promising carriers for the controlled delivery of volatile bioactive compounds, offering long‐term antimicrobial protection with potential applications in healthcare and food preservation. However, future work should address limitations, such as the moderate oil loading efficiency and the relatively complex synthesis process, to enhance scalability and practical applications. While the porous structure supports sustained release, it may also result in lower loading capacity compared to conventional adsorbents. Additionally, the multistep sol‐gel synthesis may require optimization to reduce its cost and to enhance its feasibility for large‐scale production.

Vapor‐phase antimicrobial evaluation was focused on fungi due to their significance in food‐related applications, while extension to bacterial vapor‐phase systems represents an important direction for future research. Overall, these findings highlight mesoporous C8‐silica monoliths as promising carriers for controlled, long‐term antimicrobial applications in active packaging and healthcare coatings, with future efforts directed toward enhancing oil loading capacity, simplifying fabrication, and validating performance under real‐world conditions.

## Author Contributions

W.I. designed and performed the experiments; analyzed the data related to surface characterization, thermal analysis and GC‐MS; writing—review and editing; and writing the original draft. O.I. performed the experiments. S.S. performed the experiments and analyzed the data related to antifungal activity. P.U. analyzed the data related to FTIR and TGA. T.C. performed the experiment, analyzed the data related to antimicrobial activity, writing—review and editing, and writing the original draft.

## Funding

No funding was received for this manuscript.

## Disclosure

All co‐authors revised and approved the final version of the manuscript.

## Conflicts of Interest

The authors declare no conflicts of interest.

## Supporting Information

Additional supporting information can be found online in the Supporting Information section.

## Supporting information


**Supporting Information 1** Figure S1: Photographs of C_8_‐silica monolith.

**Supporting Information 2** Figure S2: Fourier‐transform infrared (FTIR) spectrum of the C8‐functionalized silica monolith.

**Supporting Information 3** Figure S3: GC chromatogram of Eu‐oil.

**Supporting Information 4** Figure S4: GC chromatogram of Eu‐oil‐loaded C_8_‐monolith.

**Supporting Information 5** Figure S5: Inhibition zones of Eu‐oil against *Escherichia coli* and *Staphylococcus aureus* at different release times for (a) Eu‐oil on C_8_‐monolith (Day 1–Day 7). (b) Eu‐oil on filter discs (Day 1–Day 2). (c) C_8_‐monolith with DMSO (control) and (d) filter disc with DMSO (control). (e–h) Corresponding treatments against *S. aureus.*

**Supporting Information 6** Figure S6: Morphological features of *Aspergillus* sp. isolate (a) dark gray conidia with white margin, (b) cream reverse surface, (c) unbranched hyaline conidiophores (light microscopy, 100×), and (d) biseriate conidiophores with radiating phialides (400×).

**Supporting Information 7** Table S1: BET and pore structure analysis of C8‐functionalized silica monolith.

**Supporting Information 8** Table S2: Linear regression parameters for kinetic models fitted to Eu‐oil evaporation data from C_8_‐functionalized silica monolith.

## Data Availability

All data generated or analyzed during this study are included in this published article and its supporting information files.
