# Supplementary figures and images for: Prolonged Antimicrobial Effects of Eucalyptus Oil via C8‐Functionalized Silica Monolith
Source: Int J Microbiol. 2026 Jun 9;2026:6874990. doi: 10.1155/ijm/6874990 (PMC13248520; doi:10.1155/ijm/6874990)

## Slide 1
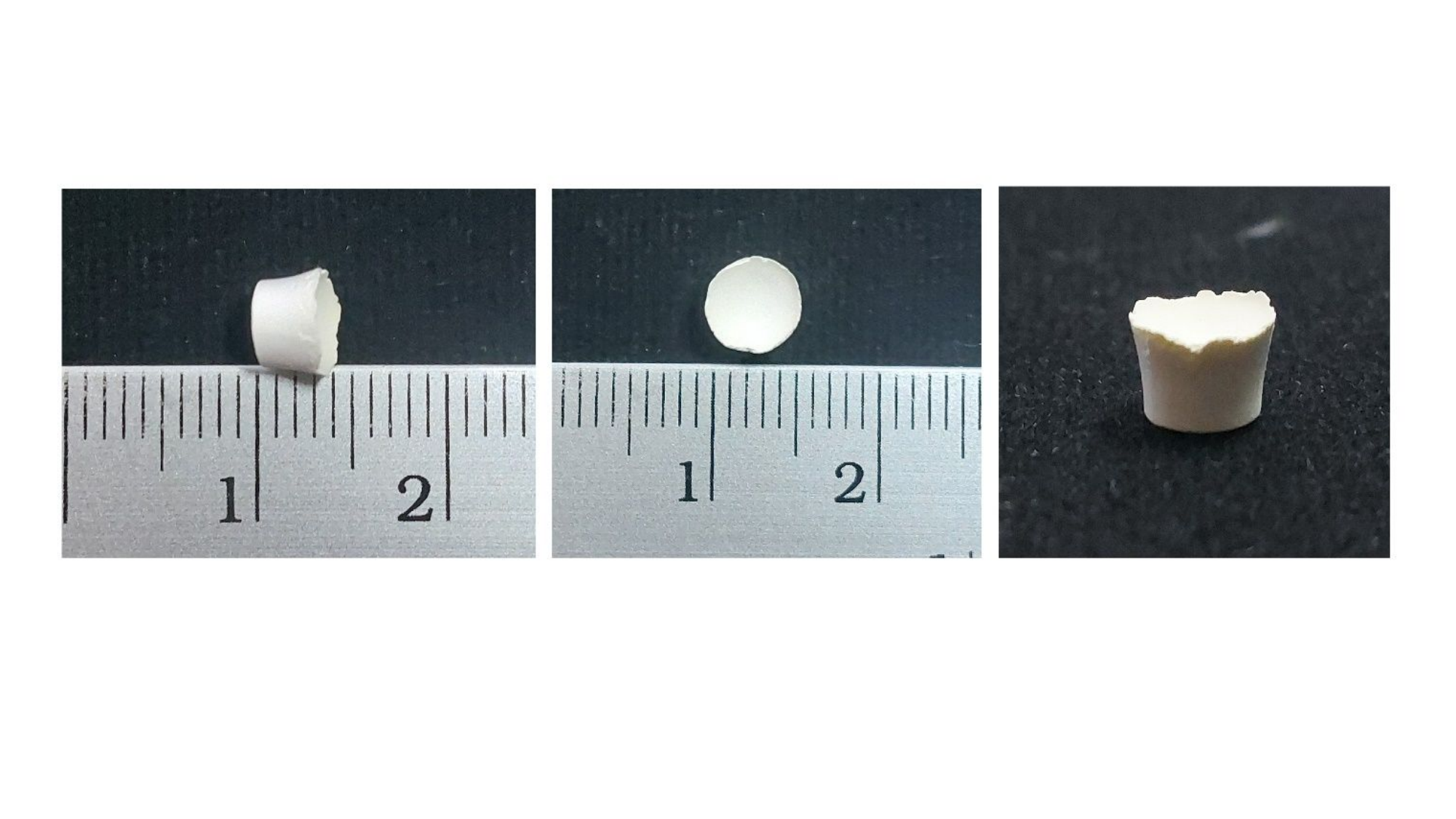

Supplement: Supplementary file 1 — Supporting Information 1 Figure S1: Photographs of C8‐silica monolith. [file IJM-2026-6874990-s007.pptx]

## Slide 1
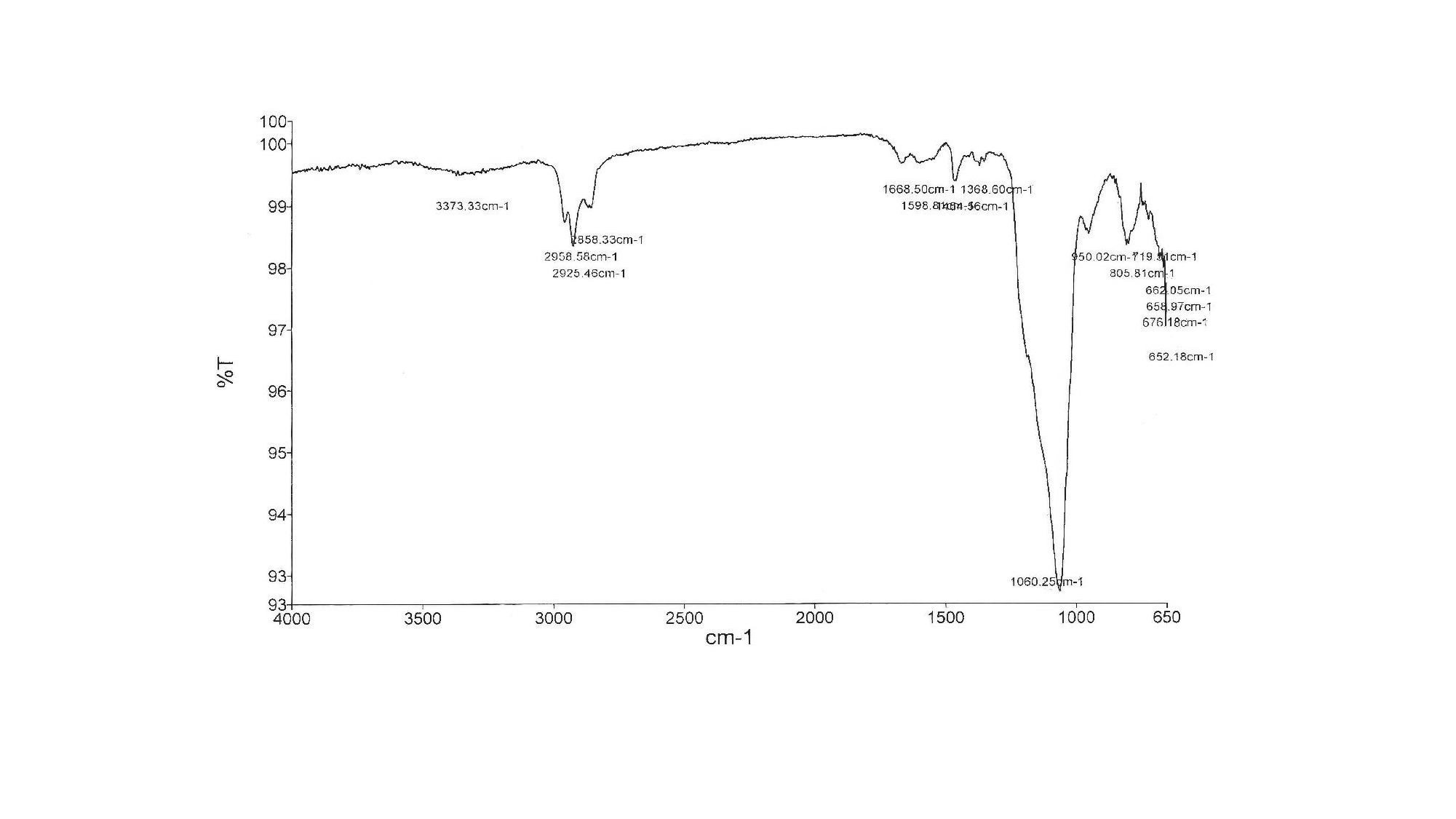

Supplement: Supplementary file 2 — Supporting Information 2 Figure S2: Fourier‐transform infrared (FTIR) spectrum of the C8‐functionalized silica monolith. [file IJM-2026-6874990-s001.pptx]

## Slide 1
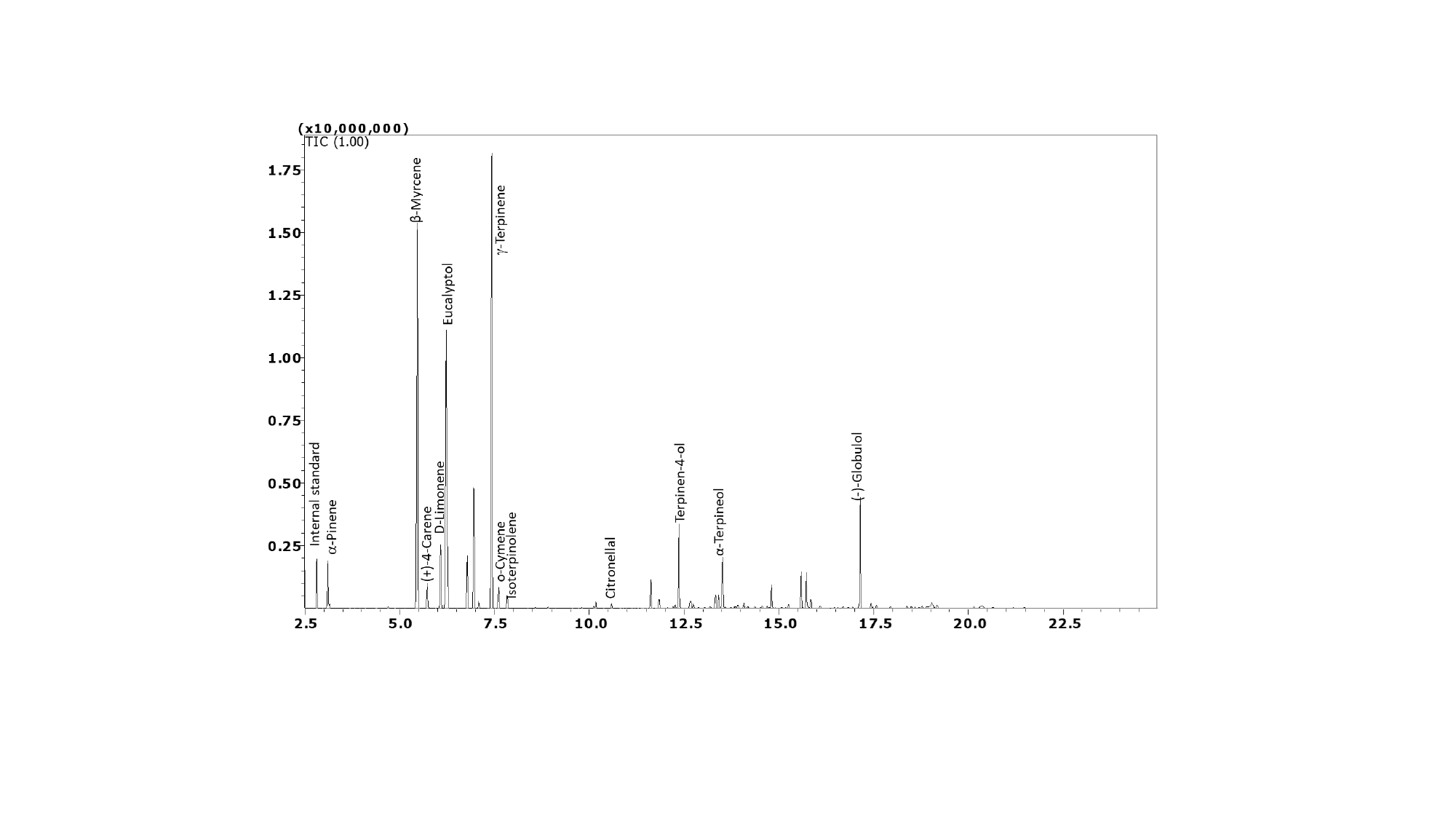

Supplement: Supplementary file 3 — Supporting Information 3 Figure S3: GC chromatogram of Eu‐oil. [file IJM-2026-6874990-s002.pptx]

## Slide 1
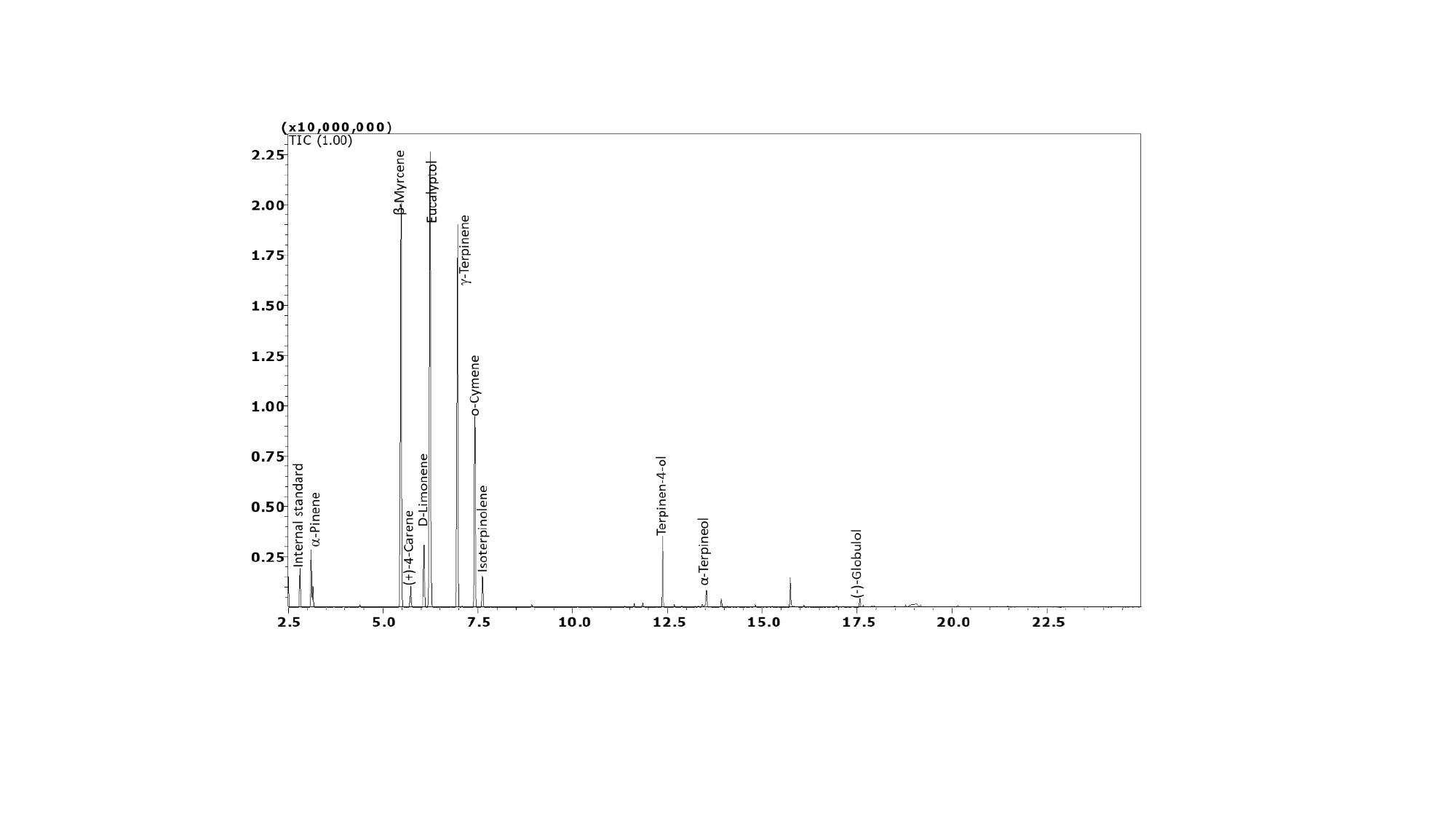

Supplement: Supplementary file 4 — Supporting Information 4 Figure S4: GC chromatogram of Eu‐oil‐loaded C8‐monolith. [file IJM-2026-6874990-s008.pptx]

## Slide 1
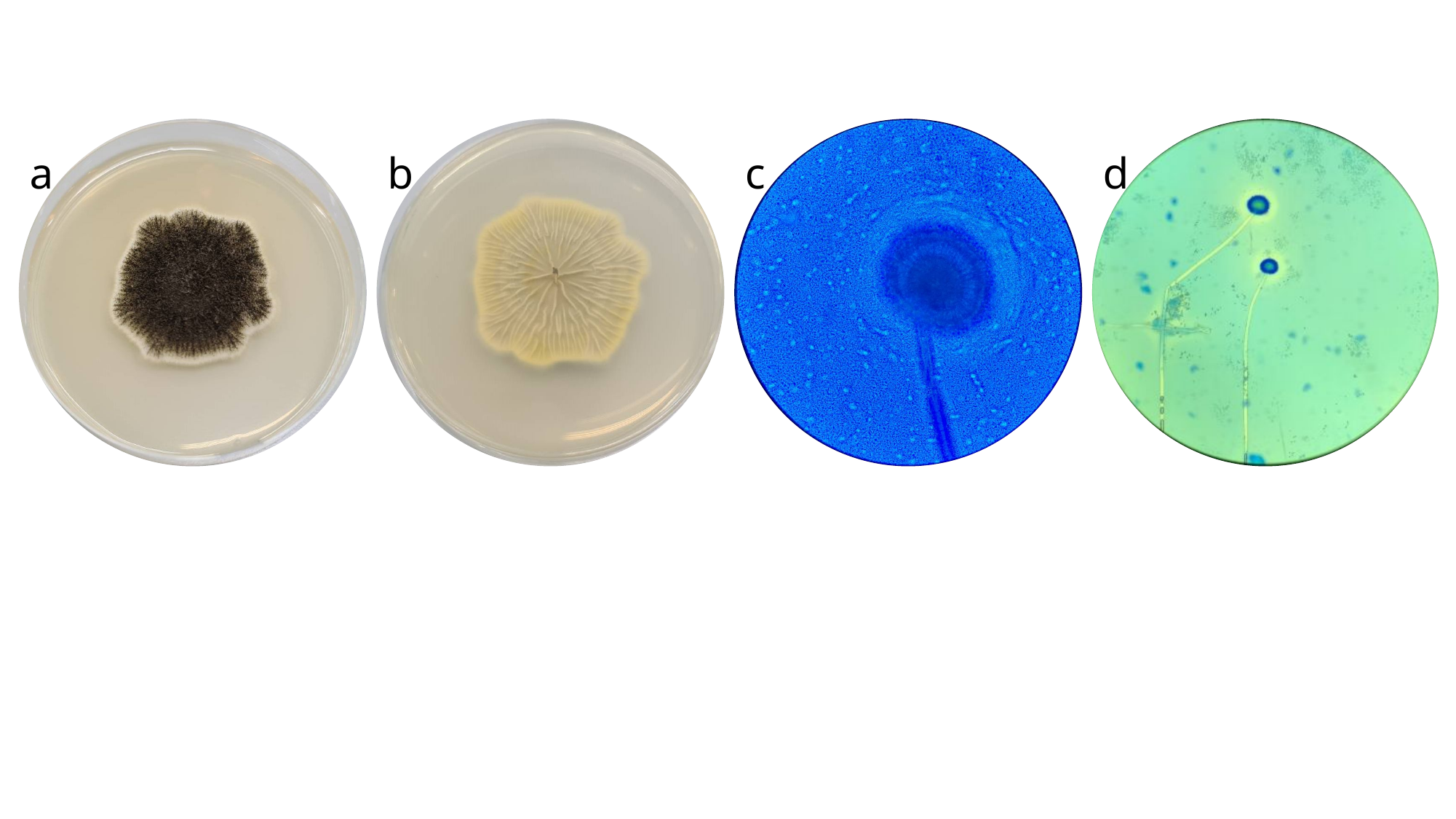

a
b
c
d

Supplement: Supplementary file 6 — Supporting Information 6 Figure S6: Morphological features of Aspergillus sp. isolate (a) dark gray conidia with white margin, (b) cream reverse surface, (c) unbranched hyaline conidiophores (light microscopy, 100×), and (d) biseriate conidiophores with radiating phialides (400×). [file IJM-2026-6874990-s005.pptx]
