# Supplementary material for: Prolonged Antimicrobial Effects of Eucalyptus Oil via C8‐Functionalized Silica Monolith
Source: Int J Microbiol. 2026 Jun 9;2026:6874990. doi: 10.1155/ijm/6874990 (PMC13248520; doi:10.1155/ijm/6874990)
Supplement: Supplementary file 5 — Supporting Information 5 Figure S5: Inhibition zones of Eu‐oil against Escherichia coli and Staphylococcus aureus at different release times for (a) Eu‐oil on C8‐monolith (Day 1–Day 7). (b) Eu‐oil on filter discs (Day 1–Day 2). (c) C8‐monolith with DMSO (control) and (d) filter disc with DMSO (control). (e–h) Corresponding treatments against S. aureus. [file IJM-2026-6874990-s004.pptx]

## Slide 1
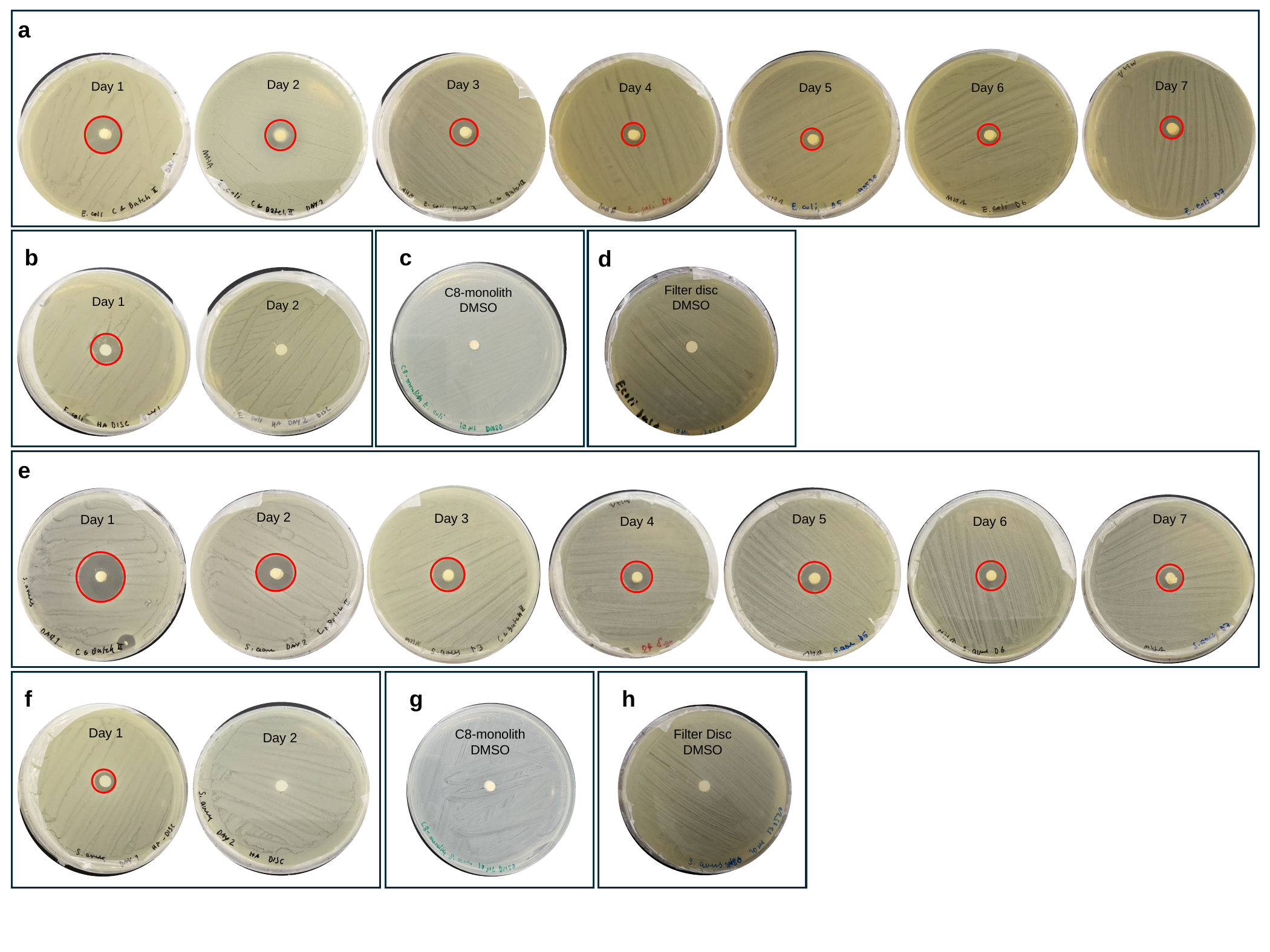

a
Day 6
Day 5
Day 7
Day 2
Day 3
Day 4
Day 1
b
c
d
C8-monolith
DMSO
Filter disc
DMSO
Day 2
Day 1
e
Day 3
Day 5
Day 1
Day 2
Day 4
Day 6
Day 7
h
f
g
Day 2
Day 1
C8-monolith
DMSO
Filter Disc
DMSO
