# Supplementary material for: Prolonged Antimicrobial Effects of Eucalyptus Oil via C8‐Functionalized Silica Monolith
Source: Int J Microbiol. 2026 Jun 9;2026:6874990. doi: 10.1155/ijm/6874990 (PMC13248520; doi:10.1155/ijm/6874990)
Supplement: Supplementary file 7 — Supporting Information 7 Table S1: BET and pore structure analysis of C8‐functionalized silica monolith. [file IJM-2026-6874990-s006.docx]

**Table S1** BET and pore structure analysis of C8-functionalized silica monolith

| **Parameter** | **Value** | **Method** |
| --- | --- | --- |
| BET surface area | 3.90 m²/g | BET |
| BJH pore diameter/size | 8.48 nm | BJH desorption |
| BJH pore volume | 0.0088 cm³/g | BJH desorption |
| DFT total pore volume/Total volume in pores | 0.00834 cm³/g | DFT |
| Median pore width | 0.99 nm | Horvath-Kawazoe |

BET = Brunauer–Emmett-Teller method for surface area analysis; BJH = Barrett-Joyner-Halenda method for pore size and volume analysis based on desorption isotherms; DFT = Density Functional Theory model for pore volume distribution; Horvath-Kawazoe = method for calculating micropore width based on adsorption potential theory.
