# Supplementary material for: Prolonged Antimicrobial Effects of Eucalyptus Oil via C8‐Functionalized Silica Monolith
Source: Int J Microbiol. 2026 Jun 9;2026:6874990. doi: 10.1155/ijm/6874990 (PMC13248520; doi:10.1155/ijm/6874990)
Supplement: Supplementary file 8 — Supporting Information 8 Table S2: Linear regression parameters for kinetic models fitted to Eu‐oil evaporation data from C8‐functionalized silica monolith. [file IJM-2026-6874990-s003.docx]

**Table S2** Linear regression parameters for kinetic models fitted to Eu-oil evaporation data from C_8_-functionalized silica monolith.

| **Model** | **Linear Equation** | **Parameter (fitted)** | **R²** | **RMSE** |
| --- | --- | --- | --- | --- |
| Zero-order | M*_t_*​ = k_0_​*t* + C | k_0_ = 15.22 | 0.9677 | 1.2934 |
| First-order | M*_t_*​ = M_α​_(1-e^-k^_1_*^​t^*) | k_1_ = 0.27 | 0.9922 | 0.0186 |
| Second-order | M*_t_*​ = (M_α_​k_2_*t* )/(1+k_2_*​t*)​ | k_2_ = 0.0055 | 0.9552 | 0.0011 |
| Higuchi | M*_t_*​ = k_H_$\surd$​*t​* | k_H_ = 47.27 | 0.9848 | 1.1739 |
| Weibull | ln[-ln(1-M_t_/100)] = b ln(*t*) + c | b = 1.30 | 0.9844 | 0.0419 |

M_t_ = cumulative amount of Eu-oil released at time t; M_α_ = total amount released at equilibrium; k_0_, k_1_, k_2_, k_H_ = release rate constants for zero-order, first-order, second-order, and Higuchi models, respectively; C = intercept in the zero-order model; R^2^ = coefficient of determination indicating goodness of fit; RMSE = root mean square error indicating model prediction accuracy; Weibull model parameter (b) = shape factor; values of b > 1 suggest non-Fickian release behavior.
